# Evaluation of the ^177m^Lu-concentration in *in-house* produced ^177^Lu-radiopharmaceuticals and commercially available Lutathera^®^

**DOI:** 10.1186/s41181-023-00222-2

**Published:** 2023-11-06

**Authors:** Matthias Balzer, Fleur Spiecker, Stephanie Bluemel, Holger Amthauer, Winfried Brenner, Sarah Spreckelmeyer

**Affiliations:** Charité - Universitätsmedizin Berlin, corporate member of Freie Universität Berlin, Humboldt-Universität zu Berlin, and Berlin Institute of Health, Department of Nuclear Medicine, Augustenburger Platz 1, Berlin, 13353 Germany

**Keywords:** Lutetium-177 m, Radionuclidic impurities, Metastable, Long-lived, Radiopharmaceutical quality control, Waste management

## Abstract

**Background:**

^177^Lu-radiopharmaceuticals can contain the metastable impurity [^177m^Lu]lutetium with a physical half-life of 160.4 days, in varying concentrations depending on the route of production of the radionuclidic precursor [^177^Lu]lutetium. Due to the long half-life of [^177m^Lu]lutetium, difficulties with waste disposal or sterility testing could arise. Here, we analyzed several ^177^Lu-samples of different origins and suppliers regarding their ^177m^Lu-concentration.

**Results:**

All samples tested showed a ^177m^Lu-concentration in the range that was stated on the certificate of analysis from the supplier which is in accordance with the European Pharmacopoeia.

**Conclusions:**

Although all ^177m^Lu-concentrations were in accordance with the European Pharmacopoeia, we need to take into account the respective national legislation regarding radioactivity release limits. With regard to the German legislation, several probes for sterility testing in external laboratories could not be released for transport due to the concentration of [^177m^Lu]lutetium. Moreover, waste water tanks should specifically be monitored for ^177m^Lu-concentration, when e.g. Lutathera^®^ is administered in the clinic.

## Background

Radioligand therapy (e.g. somatostatin analogues or protatic-specific membrane antigen (PSMA)-ligands) with the beta emitter [^177^Lu]lutetium has increased dramatically in recent years. Not only the approval of Lutathera^®^ in 2019 and Pluvicto^®^ in 2022, but also the increasing number of *in-house* productions of [^177^Lu]Lu-DOTA-TOC/TATE or [^177^Lu]Lu-PSMA ensures an increasing demand for approved [^177^Lu]LuCl_3_ as precursor. According to the German Medicines Act, radionuclides as precursors for radiopharmaceuticals must be approved. In January 2022, two suppliers have been approved in Germany to distribute [^177^Lu]lutetium, namely IDB Holland (Netherlands) and ITM Isotope Technologies (Germany). According to the European Pharmacopoeia, [^177^Lu]lutetium for radiolabeling must have a radionuclidic purity of greater than 99%, whereas [^176^Yb]ytterbium (< 0.1%), [^177m^Lu]lutetium (< 0.07%) and an overall radionuclidic impurity (without [^176^Yb]ytterbium and [^177m^Lu]lutetium) of < 0.01% is allowed. In addition, radiopharmaceuticals for i.v. application must be sterile and therefore tested for sterility. In most radiopharmacies, producing in-house radiopharmaceuticals, the samples for sterility testing are sent to an external laboratory for analysis. The samples for sterility are therefore transported directly after synthesis, labeled as radioactive transport, to the external laboratory, or—as in most radiopharmacies—the samples are sent after the radioactivity reached the exemption limit. For [^177m^Lu]lutetium, the exemption limit is 1 × 10^6^ Bq and 0.1 Bq/g for the unrestricted release of solid and liquid substances (Strahlenschutzverordnung Anlage 2018).

[^177^Lu]lutetium decays to stable [^177^Hf]hafnium with a physical half-life of 6.64 days. The emitted beta particles have a maximum energy of 497 keV (79%) and low energy low abundance gamma rays of 208 keV (10.4%) and 113 keV (6.2%). The long-lived [^177m^Lu]lutetium decays in 22.7% of the cases to [^177^Lu]lutetium and in the other cases to [^177^Hf]hafnium (77.3%). In the first case the main gamma emission is 414 keV (17.4%). In the second case the main gamma emissions are 419 keV (21.7%), 379 (29.4%), 228 keV (35.9%), 208 keV (55.4%) and 113 keV (21.4%).

**Fig. 1:**
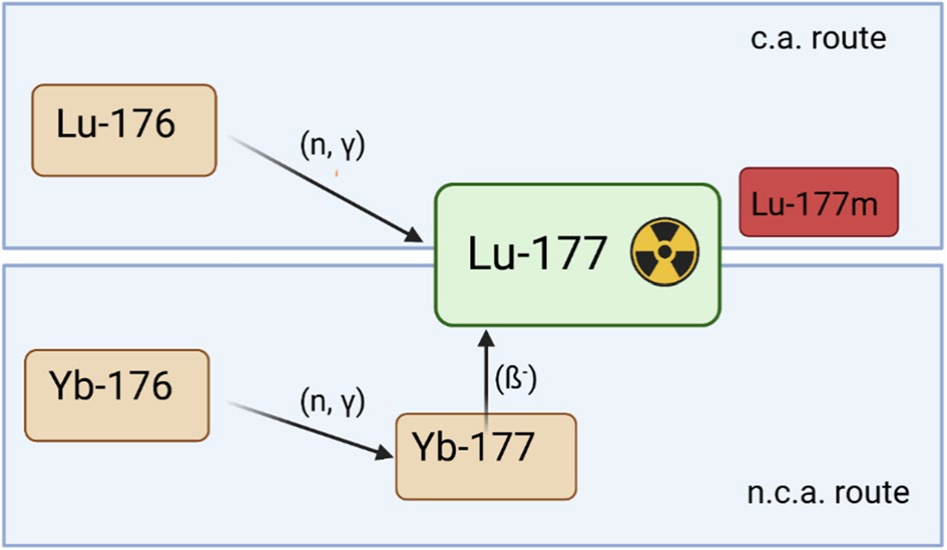
Production routes for lutetium-177^2^, created with biorender.com

For the reactor production of [^177^Lu]lutetium, two routes are possible—either non-carrier added (n.c.a) or carrier-added (c.a.), Fig. 1.

The n.c.a. pathway leads via neutron irradiation of [^176^Yb]ytterbium to [^177^Yb]ytterbium, which then decays to [^177^Lu]lutetium by beta emission, ^176^Yb(n,γ)^177^Yb → ^177^Lu. The target material [^176^Yb]ytterbium is enriched to a level above 99% (Barkhausen 2011). The direct route leading from neutron irradiation of [^176^Lu]lutetium to [^177^Lu]lutetium, ^176^Lu(n,γ)^177^Lu, leads to the by-product of [^177m^Lu]lutetium (physical half-life 160.4 days).

The first approved drug that contains [^177^Lu]lutetium is Lutathera^®^. There are already several publications that describe the presence of ^177m^Lu-impurities in Lutathera^®^ (Brown 2020; Prevot et al. 2023). The problem that might arise in some nuclear medicine facilities is related to the exemption limit of 5 × 10^5^ Bq/m^3^ in the waste water (Strahlenschutzverordnung Anlage 2018). If this limit is reached it would result in waste management problems like longer storage times. Worst case, if the contaminated water would enter unknowingly into waste water systems and could be harmful to the environment. Due to the recent approval of Pluvicto^®^, an increased demand for [^177^Lu]lutetium is assumed. Since nuclear medicine departments require an uninterrupted, stable, reliable and sufficient supply with [^177^Lu]lutetium (Vogel et al. 2021), several additional suppliers of [^177^Lu]lutetium might enter the market soon.

A recent study by Prevot et al. demonstrated that 45 vials of administered Lutathera^®^ contained 0.3% [^177m^Lu]lutetium (Prevot et al. 2023). The authors claim a waste disposal plan that anticipates a minimum of three years storage in terms of empty vials and biohazards (less than 1 MBq ^177m^Lutetium-content and less than 0.1 µSv/h at contact) and five years for partially filled vials.

Freudenberg et al. evaluated the ^177m^Lutetium-content in Lutathera^®^ (n = 4) and EndolucinBeta^®^ (n = 6) probes (Freudenberg et al. 2022). They concluded that Lutathera^®^ has an average concentration of 311 Bq/g ± 200 Bq/g and 0.8 ppm [^177m^Lu]lutetium. For EndolucinBeta^®^, they found an average of 1.4 Bq/g and 0.0024 ppm [^177m^Lu]lutetium.

In order to be able to estimate the possible effects of further approvals of ^177^Lu-therapeutics on ^177m^Lu-concentrations in waste water, we evaluated 97 retention samples of ^177^Lu-therapeutics that were produced in-house with approved EndolucinBeta. Furthermore, we evaluated the effect of [^177m^Lu]lutetium impurities on the realization of sterility controls as required by Ph. Eur. for sterile radiopharmaceuticals for i.v. application. In addition, we performed test synthesis with other [^177^Lu]lutetium suppliers, i.e. IDB Holland, Eckert & Ziegler Radiopharma GmbH (EZAG), Novartis Pharma GmbH and Monrol Nuclear Products Co and analyzed the samples towards their ^177m^Lu-concentrations. Additionally, we analyzed 38 Lutathera^®^ probes.

## Results

**Fig. 2:**
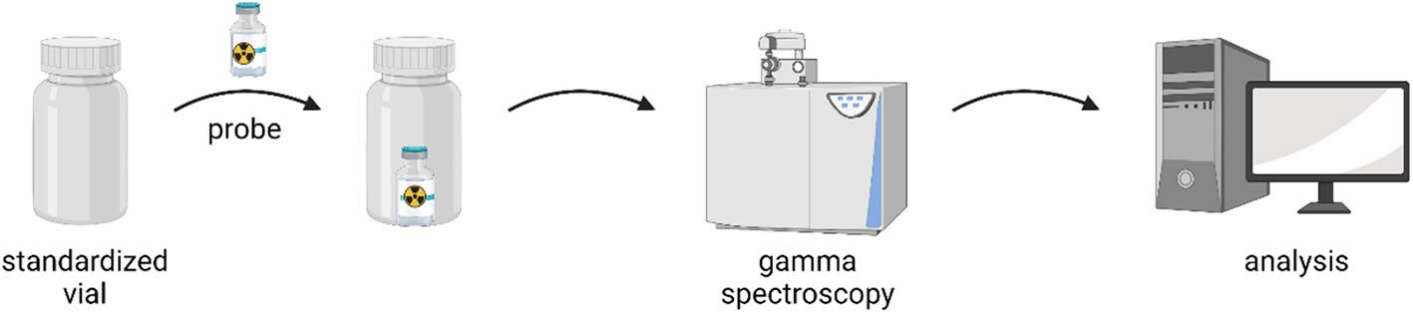
Schematic overview of experimental setup, created with biorender.com

**Fig. 3:**
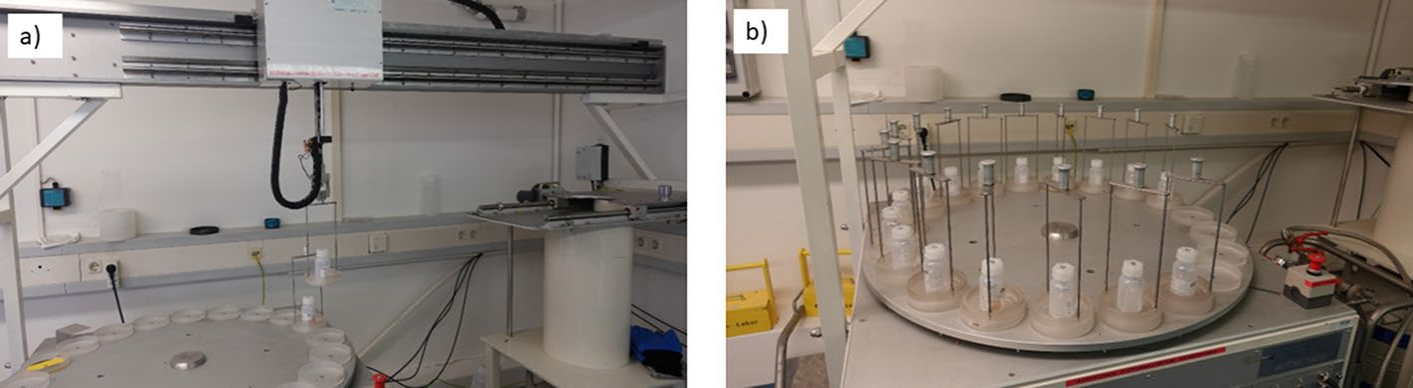
Gamma spectrometer **a** full view of automatic sample changer (left) sample arm (middle) and lead shielded detector (right) and **b** sample tray

The experiments were performed following the schematic protocol described in Fig. 2 and Fig. 3. In total, 138 samples from year 2021 were analyzed by gamma spectroscopy and analyzed with the program Apex-Gamma (Version 1.4.1) for their ^177m^Lu-content. The results obtained were normalized by recalculation to the activity and volume on the calibration date. For the retention samples, the exact amount of [^177m^Lu]lutetium in the volume in the retention sample was calculated.

### ***EndolucinBeta***^®^*** (probes 1–97)***

**Fig. 4.**
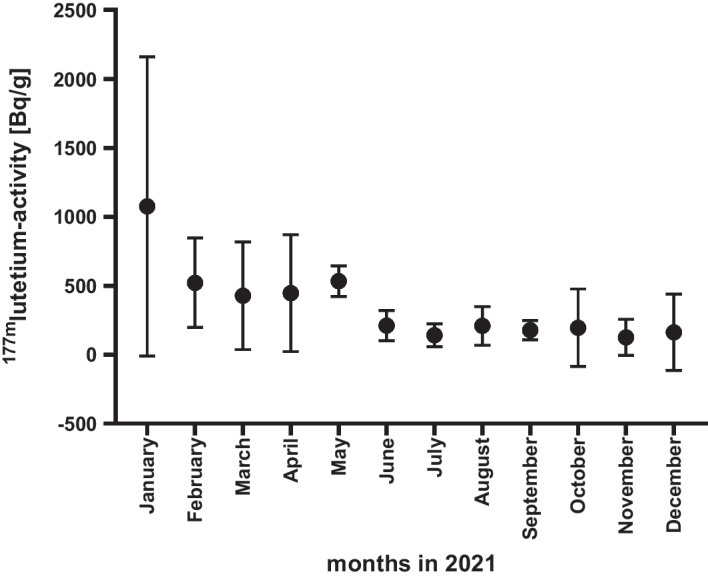
^177m^Lu-activity found in months of 2021

Figure 4 shows the ^177m^Lu-activity that was found in the EndolucinBeta^®^ (ITM) samples in the respective months of the year 2021 (January until December). In January, we measured an ^177m^Lu-activity of 1075 Bq/g. From February until May, we measured an ^177m^Lu-activity of approximately 482 Bq/g and from June until December, the ^177m^Lu-activity was around 175 Bq/g. The mean ^177m^Lu-activity in the samples of EndolucinBeta^®^ was 381 ± 511 Bq/g, which represents a radionuclidic impurity of <  < 0.01% (approx. 0.0000037 ± 0.0000053%) which is very far below the limit of 0.01% stated on the certificate of analysis (see Table 1).

**Table 1:** Overview of suppliers of tests Lu177-precursors; * = ITM, ** = EZAG, *** = Monrol

Probe number	Supplier	Lu177m-impurity on certificate of analysis (%)	Production route	Activity of Lu177m [Bq/g] d.c	Activity of Lu177m [ppm] d.c	Activity of Lu177m [%] d.c	Measurement error [%]
0	1	2	3	4	5	6	7
1–97	EndolucinBeta^®^*	< 0.01	n.c.a	381 ± 511^a^	0.00037 ± 0.00053^a^	0.0000037 ± 0.0000053^a^	≤ 4.7
98	^177^Lu Lutetiumchloride in aqueous solution **	< 0.007	n.c.a	9521	0.00453	0.0000453	≤ 3.9
99	Lutetium chloride (Lu-177) radiopharmaceutical precursor solution nca ***	not detected	n.c.a	39	0.00002	0.0000002	≤ 3.7
100	LuMark^®^	0.00786	c.a	706,594	0.82479	0.008248	≤ 3.0
101–138	Lutathera^®^ (n = 38, n = 25 for column 4)	< 0.015	c.a	29,512 ± 6491^a^	0.96 ± 0.17^a^	0.0096 ± 0.0017^a^	≤ 2.6

^a^Standard Deviation

Regarding waste management, we evaluated the volume in the retention samples of the EndolucinBeta^®^ probes for their ^177m^Lu-content. Here, we observed a ^177m^Lu-content of 61 ± 101 Bq/g.

### ***Non-approved [***^***177***^***Lu]lutetium (probes 98 and 99)***

For the test syntheses with non-approved [^177^Lu]lutetium probes at the date of testing (EZAG and Monrol), we found 9521 Bq/g (± 371 Bq/g with an measurement error of 3.9%) which results in a radionuclidic impurity of <  < 0.01% (approx. 0.0000453%) of [^177m^Lu]lutetium for EZAG and 39 Bq/g (± 1 Bq/g with an measurement error of 3.7%) and <  < 0.01% (approx. 0.0000002%) of [^177m^Lu]lutetium for Monrol (see Table 1). Both values are very far below the stated limits on the certificate of analysis.

### ***LuMark***^®^*** (probe 100)***

For the test synthesis with approved LuMark^®^ (IDB Holland), we found 706,594 Bq/g (± 21,198 Bq/g with an measurement error of 3.0%) resulting in radionuclidic impurity of < 0.01% (approx. 0.008248%) of [^177m^Lu]lutetium which is in the range of the stated ^177m^Lu-content on the certificate of analysis, when the measurement error of 3.0% is taken into account.

### ***Lutathera***^®^*** (probes 101–138)***

For Lutathera^®^ (Novartis), we analyzed a total of 38 samples—29 empty Lutathera^®^ vials and 9 samples with residual activity that was filled into extra vials before the patient received a reduced Lutathera^®^ activity. We found 29,512 ± 6491 Bq/g [^177m^Lu]lutetium in Lutathera^®^, corrected to the time of the first measurement before application, resulting in an radionuclidic impurity of < 0.01% (approx. 0.0096%) of [^177m^Lu]lutetium. Novartis Pharma GmbH replied to our inquiry that the ratio of [^177m^Lu]lutetium in Lutathera^®^ is below 0.015% and the absolute [^177m^Lu]lutetium-activity is about 1 MBq.

## Discussion

We analyzed 97 EndolucinBeta^®^ probes regarding their ^177m^Lu-content via gamma spectroscopy. First, we evaluated the results with respect to the stated ^177m^Lu-impurity on the certificate of analysis. The specifications and measured activities of [^177m^Lu]lutetium were within the criteria limits and the stated values were successfully reproduced. Secondly, we evaluated the ^177m^Lu-content and consequences for sterility testing or waste disposal with regard to the retention samples. The retention samples of EndolucinBeta^®^ (n = 97), showed an average ^177m^Lu-content of 61 Bq/g ± 101 Bq/g in a volume ranging from 0.4 mL to 11.1 mL. With a physical half-life of 160.4 days, it will take approximately 5 years until the ^177m^Lu-content undercuts the exemption limit of 0.1 Bq/g. Consequently, the retention samples for sterility testing would need to be transported with a pricy radioacitivity transport to an external laboratory for analysis as well as the laboratory must have a permit for handling. To prevent this, an in-house sterility testing might need to be established. In 13 out of 97 probes, no ^177m^Lu-content was detected. The storage period for retention samples of radiopharmaceuticals is 6 months according to GMP guidelines (Commission 2008). Thus, an extra 4.5 years is necessary until the retention samples can be safely disposed.

The other three ^177^Lu-samples from different suppliers (EZAG, Monrol, IDB Holland) were analyzed regarding their ^177m^Lu-content without patient application. To note, for each supplier, we only had one probe (n = 1) in contrast to EndolucinBeta^®^ (n = 97). As seen in Table 1, Monrol had the lowest ^177m^Lu-content of 39 Bq/g and 0.00002 ppm of the total ^177^Lu-activity of 21.21 GBq. On the other hand, the c.a. production from IDB Holland clearly showed an elevated concentration of [^177m^Lu]lutetium. Here, we found 0.8 ppm of [^177m^Lu]lutetium.

For Lutathera^®^, we found higher ^177m^Lu-concentrations compared to n.c.a. produced [^177^Lu]lutetium. In 38 analyzed samples, we stayed within the Ph. Eur. required ^177m^Lu-activity level of 0.07%.

## Conclusions

We can conclude, that the measured ^177m^Lu-impurity concentrations of all suppliers were within the limits stated on the respective certificate of analysis. We also detected [^177m^Lu]lutetium in n.c.a. ^177^Lu-samples. This can be explained by the target material, which is usually > 99%, but not 100% pure. Regarding the elevated ^177m^Lu-content in Lutathera^®^ and LuMark^®^, each nuclear medicine facility and radiopharmacy needs to pay very close attention to a safe waste disposal and shipping for e.g. sterility testing with respect to their local and national radiation safety regulations and radioactivity release limits. We did not include Pluvicto^®^ in our studies, as there was no sample available in our institution. But we would like to highlight, that in the smPC of Pluvicto^®^ it is stated, that the radioactive precursor might either produced by the direct or the indirect route (smPC. of Pluvicto 2022) and that special attention should be drawn to the respective batch release certificate. No such comment was found in the smPC of Lutathera^®^ (smPC. of Lutathera 2022).

## Methods

### Probes

The probes originate from in-house- or validation productions of ^177^Lu-radiopharmaceuticals. The [^177^Lu]LuCl_3_ used as starting material, was purchased either from ITM Garching (EndolucinBeta^®^, Probe 1–97, calibrated in year 2021), Eckert and Ziegler (probe 98, calibration date 25. 02. 2021), Monrol (probe 99, calibration date 15. 03. 2022) or IDB Holland (probe 100, calibration date 19. 06. 2018). Additionally, we analyzed 38 probes from Lutathera^®^ (probe 101–138).

All probes were decaying for at least 85 days so that the ^177^Lu content is low enough to measure metastable contamination with gamma spectroscopy.

### Gamma spectroscopy

Gamma radiation was measured for 60 min with deadtime correction using a standard coaxial *Germanium-detector* [HPGe detector, GC2020, Mirion Technologies (Canberra) GmbH]. The output is connected to a LYNX multichannel analyzer. The measured spectra were analyzed with the program Apex-Gamma (Version 1.4.1) for their ^*177m*^Lu-content. The *analysis program* calculates the activity from the measured energy peaks, the probability of emission and the energy-dependent efficiency. In the report it lists the mean activity of all analyzed energy peaks, which are given in the nuclide library. The report also shows the measurement error. It is multifactorial and includes the uncertainty of the instrument, the uncertainty of the Poisson statistics and the differences between the activities calculated from the individual peaks. The measurement is limited by the influence of residual ^*177*^Lu, which affects the activity calculation of ^*177m*^Lu. However, since the energy spectrum of the two nuclides differs by the energy 228 keV, which is only emitted by ^*177m*^Lu, this error is also represented by the listed measurement errors (Table 1), since they include the scatter of the calculated activities of the different energy peaks. The detection limit is depending on the background and the nuclide, for the measured Lutathera Vials the mean detection limit for ^*177m*^Lu is 29.8 ± 22.2 Bq (values reach from 12.8 Bq to 94.1 Bq). To ensure the reproducibility of the gamma spectroscopy measurements monthly quality assurances are performed. Data was recorded and stored on a APEX PC, Windows 10 Pro (2019) and processed and analyzed with Apex Gamma software.

In our experiments, we used *correction factors* to increase the accuracy of our measurements. Only the 100 ml Kautex vials are calibrated on our system. Consequently, we had to calculate a correction factor for each different vial type. First, the probes were analyzed as described below. Secondly, the probe (e.g. 27 mL vial) was rinsed with approximately 50 mL of demineralized water and refilled up to 100 mL into a 100 mL plastic Kautex vial and the activity was measured by gamma spectroscopy. For the 27 mL Lutathera^®^ vial (n = 29), we calculated a correction factor of 0.66 (the vials with the reduced dose had a correction factor of 0.55). For EndolucinBeta (n = 97), IDB Holland (n = 1) and EZAG (n = 1), the correction factor was 0.48.

From 34 received batches Lutathera^®^ (in 52 vials), we used the released documents to calculate the amount of [^177m^Lu]lutetium according to the manufacturer's measurements to 0.0087% ± 0.0019% of the total activity or 28,063 ± 5884 Bq/g at the planned time of injection.

## Abbreviations

PSMA: Prostate-specific membrane antigen
ppm: Parts per million
Ph. Eur.: European Pharmacopoeia
GMP: Good manufacturing practice
smPC: Summary of product characteristics

## Declarations

### Availability of data and materials

The datasets used and/or analysed during the current study are available from the corresponding author on reasonable request.
